# HLA B27 antigen in Middle Eastern and Arab countries: systematic review of the strength of association with axial spondyloarthritis and methodological gaps

**DOI:** 10.1186/s12891-017-1639-5

**Published:** 2017-06-29

**Authors:** Nelly Raymond Ziade

**Affiliations:** 1 Rheumatology Department, Saint-Joseph University, Beirut, Lebanon; 2 Rheumatology Department, Hotel-Dieu de France Hospital, Beirut, Lebanon; 3 Tour des Consultations Externes, Hotel-Dieu de France hospital, 6th floor, Alfred Naccache blvd, Achrafieh, PO BOX 166830, Beirut, Lebanon

## Abstract

**Background:**

Axial spondyloarthritis (AxSpA) is a relatively frequent and debilitating disease, with a prevalence ranging from 0.1 to 2% in the Caucasian population. Current Assessment of Spondyloarthritis International Society (ASAS) classification criteria of AxSpA rely either on sacroiliitis on imaging plus one SpA feature or positive HLAB27 antigen plus two SpA features, in a patient with chronic low back pain and age at onset of less than 45 years. Therefore, HLA-B27 is a central feature in SpA classification and plays a pivotal role in referral strategies and early diagnosis. The primary objective of the study is to review the prevalence of HLA-B27 in normal and AxSpA populations in Middle Eastern and Arab Countries and to assess the strength of association between HLA-B27 antigen and AxSpA. The secondary objective is to identify the gaps in the methodology of the studies and suggest a framework for future research.

**Methods:**

Studies were included in the analysis if they reported prevalence of HLA-B27 in AxSpA and/or general population and if they covered geographical location in the Middle East or Arab countries in the Mediterranean basin. Odds ratios (OR) were calculated for each country, as a measure of the strength of association between HLA-B27 and AxSpA, compared to the normal population, using the two-by-two frequency table. Available data from the literature were analyzed according to the following quality indicators: sample size, method of HLA-B27 testing, presence of control group and external validity.

**Results:**

Twenty-seven studies were analyzed. HLAB27 prevalence in the normal population ranged from 0.3% (Oman) to 6.8% (Turkey). HLA-B27 prevalence in AxSpA ranged from 26.2% (Lebanon) to 91% (Turkey). HLA-B27 prevalence in all SpA ranged from 13.87% (Lebanon) to 69.43% (Kuwait). Peripheral SpA was less associated with HLA-B27 than AxSpA, indicating the need of differentiating between the two entities when calculating prevalence. When available (8 studies), the OR ranged from 21.63 (Morocco) to 105.6 (Syria). The high heterogeneity between the results can be due to differences in methodology: study sample size, different classification criteria, absence of control groups, HLA-B27 testing method.

**Conclusions:**

The prevalence of HLA-B27 in the normal population is significantly lower in the Middle Eastern and Arab countries than in Western Countries. However, HLA-B27 testing can be useful for AxSpA positive diagnosis, given the high OR. Heterogeneity between countries may be due to methodological differences.

## Background

Axial spondyloarthritis (AxSpA) is a relatively frequent disease, with a prevalence ranging from 0.1 to 2% in the Caucasian population. It affects mainly young adults and can be significantly disabling with serious professional impact and high socioeconomic cost [1, 2]. Although AxSpA is a chronic disease evolving over several decades, recent evidence shows that early diagnosis and treatment may lead to better outcome [3]. Over the years, many classification systems were used to diagnose and classify spondyloarthritis [4–6], aiming, in the most recent Assessment of Spondyloarthritis International Society (ASAS) classification criteria, to reach earlier diagnosis [7]. The current ASAS classification of AxSpA relies either on sacroiliitis on imaging plus one SpA feature (imaging arm) or HLA-B27 antigen plus two SpA features (clinical arm), in a patient with chronic low back pain and age at onset of less than 45 years [8]. Therefore, HLA-B27 plays a central role in SpA classification and can be pivotal in referral strategies and early diagnosis [9, 10]. The association of axial SpA with HLA-B27 seems to be lower in most Arab populations compared to Western European populations [11], possibly due to genetic backgrounds. This may affect the diagnostic value of HLA-B27 antigen, and impact the local application of published referral strategies, which were studied in Western populations with high HLA-B27 prevalence [9]. However, the heterogeneity in HLA-B27 prevalence within Middle Eastern and Arab Countries [12] can be related either to true differences between the populations or to heterogeneity in the methodology of the studies. This heterogeneity may lead to errors in the estimation of the disease prevalence and delay in diagnosis. Previous studies reviewed the prevalence of HLA-B27 antigen in SpA and in the general population [11], but the strength of association was not numerically assessed regardless of the prevalence. The primary objective of the study is to review the prevalence of HLA-B27 in normal and AxSpA population in Middle Eastern and Arab Countries and to assess the strength of association between the HLA-B27 antigen and AxSpA in different populations. The secondary objective is to identify the gaps in the methodology of the studies and suggest a framework for future research.

## Methods

A literature review on PubMed from beginning until nowadays was performed, using the following MeSH terms: HLA-B27 antigen, prevalence, ankylosing spondyloarthritis, spondyloarthritis, Middle East, Arab countries, in parallel with cross-reference search. Inclusion criteria were: studies evaluating the prevalence of HLA-B27 antigen (regardless of the subtype) in AxSpA and/or the general population, inclusion of SpA groups according to any of the contemporary diagnostic criteria [New York (NY), Amor, ESSG, ASAS] and geographical situation in the Middle East as well as other Arab countries of the Mediterranean basin. Data were extracted from the Internet and pertinent information from the articles were recorded in a table following pre-specified criteria. For each study, the prevalence of HLA-B27, the country, the study sample size, and the type of population were reported. For SpA, the criteria type was reported, and the type (general population, other rheumatic diseases or blood donors) was recorded for the controls. Then, the strength of association between HLAB27 antigen and spondyloarthritis was assessed using the odds ratio [OR]. The OR represents the odds that an outcome (SpA) will occur given a particular exposure (HLA-B27), compared to the odds of the outcome occurring in the absence of that exposure. ORs are most commonly used in case-control studies; however, they can also be used in cross-sectional studies similarly to those included in this review. When available, the number of exposed (a + b) and non-exposed (c + d) were extracted from the studies and computed in a two-by-two frequency table against the disease status [SpA (a + c) and controls (b + d)]. The OR is calculated using the classic formula: a*d / b*c [13]. Values greater than one indicate a positive association between HLA-B27 and Spa; higher ORs indicate a stronger association. 95% confidence intervals [CIs] were calculated for each OR when available. Furthermore, available studies were analyzed according to the following quality indicators: sample size, method of HLA-B27 testing, presence of control group and external validity. A Forest plot was constructed for ORs and 95%CIs to visually assess their heterogeneity. Statistical analysis was performed using the MedCalc software, version 17.4.

## Results

**Fig. 1:**
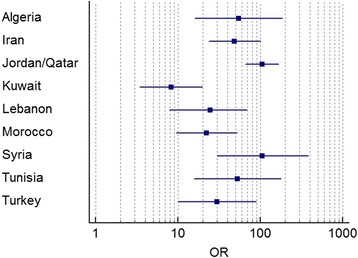
Forest plot showing the available odds ratios by country (OR, in logarithmic scale) and their respective 95% confidence intervals

Twenty-seven studies, published between 1978 and 2012, were analyzed. All studies corresponding to the pre-specified eligibility criteria were included in the review. High heterogeneity was found between prevalence rates in the different studies. HLA-B27 prevalence in the normal population ranged from 0.3% (Oman) to 6.8% (Turkey). HLAB27 prevalence in AxSpA ranged from 26.2% (Lebanon) to 91% (Turkey). HLA-B27 prevalence in peripheral SpA ranged from 13.87% (Lebanon) to 69.43% (Kuwait). Similarly to international studies, peripheral SpA was less associated with HLA B27 than AxSpA, indicating the need of differentiating between the two clinical entities when calculating prevalence (Table 1). More than two-thirds of the studies had no control groups; therefore, the measure of the strength of association was not possible in all countries, and the true diagnostic properties could not be assessed. When available (8 studies), the OR (HLA-B27 in AxSpA compared to HLA-B27 in the normal population) ranged from 21.63 (Morocco) to 105.6 (Syria). ORs are presented in Fig. 1, with their respective 95%CIs. This Forest plot indicates large CIs and heterogeneity between the studies, although all ORs were largely greater than 1. Only 11 studies had both AxSpA and control groups. About one-third of the studies had a very low sample size (8 out of 22 AS studies had less than 30 included patients). The method of HLA-B27 testing was not included in the final quality assessment since it could not be identified in most of the studies. Clear and homogeneous inclusion criteria (unique classification criteria, no mixing with other diseases or subtypes such as Behçet’s diseases or coxitis) were found in only two-thirds of the studies (14 out of 22). Most heterogeneity comes from the mixing of different types of SpA (SpA plus other forms diagnosed by ESSG criteria – which may include peripheral forms as well). The evaluation of the studies according to the pre-defined quality indicators is summarized in Table 2.

**Table 1:** Summary of the studies on HLA-B27 antigen prevalence in SpA and the normal population in Middle Eastern and Arab Countries

Country	Sample Size SpA	Sample Size (Control)	Prevalence in Population	Prevalence in SpA	Prevalence in AS	OR AS/GP [95% CI]	Reference
Algeria	129 AS (ESSG and NY)	76 healthy blood donors	4%		69%	54.14 [16.09–182.18]	[17]
Egypt		380 normal individuals	4.7%				[18]
Egypt	75 SpA (ESSG) including 34 AS			58.7%			[19]
Egypt		100 healthy controls	1%				[20]
Iraq	25 AS		2.1%		84%		[21]
Iran	60 AS	430 healthy blood donors	3.95%		66.67%	48.59 [23.57–100.17]	[22]
Iran	119 AS				68.91%		[23]
Iran	98 AS (NY)				73.4%		[24]
Israel	38 AS	456 normal individuals	3%		79%		[25]
Jordan	22 AS (NY)				75%		[26]
Jordan / Qatar	129 AS	2579 healthy individuals	2.4%		72.22%	104.87 [66.21–166.11]	[12]
Kuwait	58 SpA (27 AS)			69.43%	82.56%		[27]
Kuwait	35 SpA + AS patients	544 controls	4%	25.7%		8.21 [3.44–19.60]	[28]
Lebanon	105 SpA (ESSG) 24 AS (NY)	841 Rheumatolgy patients	1.44%	13.85%	26.32%	24.46 [7.78–68.19]	[29]
Morocco	46 AS + coxitis	183 healthy controls	6.16%		58.70%	22.09 [9.48–51.49]	[30]
Morocco	49 SpA (Amor and ESSG)				67%		[31]
Oman		321 healthy	0.3%				[32]
Qatar	119 AS (NY)				74%		[33]
Saudi Arabia	12 AS				66.67%		[34]
Syria	50 AS (NY)	217 healthy controls	1.4%		60%	105.64 [29.98–381.87]	[35]
Tunisia	365 AS and/or BD	124 controls	3.2%		42.9%		[36]
Tunisia	100 AS (NY)	100 healthy controls	3%		62%	52.75 [15.61–178.31]	[37]
Tunisia	50 AS (Amor / ESSG)				69%		[38]
Turkey	112 AS (NY)	55 controls	2.8–6.8%		70%	29.82 [9.99–89.05]	[39]
Turkey	216 AS (67 analyzed)				91%		[40]
UAE		760 healthy	6.4%				[41]
UAE	17 AS				56%		[42]

*AS* Ankylosing Spondylitis, *BD* Behcet’s disease, *CI* Confidence Interval, *ESSG* European Spondyloarthropathy Study Group analysis, *GP* General Population, *NY* New York criteria, *OR* Odds Ratio, *SpA* Spondyloarthritis

**Table 2:** Evaluation of the studies according to the quality indicators

Quality Indicator	Number of studies / Total studies
Sample size	
- AS <30 patients	8 / 22
- AS >50 patients	12 / 22
Presence of control group	11 / 27
Method of HLA-B27 testing stated	3 / 27
Homogeneous and clear inclusion criteria	14 / 22

## Discussion

The prevalence of HLA-B27 in the normal population is significantly lower in the Middle Eastern and Arab countries (0.3% to 6.8%) than rates reported in the United States and Europe (6% to 25%) [14–16]. Nonetheless, HLA-B27 seems to be correlated with AxSpA as reflected by high ORs (HLA-B27 in AxSpA compared to HLA-B27 in the normal population), and HLA-B27 testing can be useful for AxSpA diagnosis. However, the local data should be considered when adapting the published referral strategies, as those strategies were tested in countries with high HLA-B27 prevalence. Heterogeneity between Middle Eastern and Arab countries may be mostly due to methodological differences in the studies rather than to true differences between the populations. The first heterogeneity is related to SpA samples: some studies have very low sample size (as low as 12 patients), leading to large CIs indicating poorly powered studies. Also, heterogeneity may result from differences in the classification criteria used: AxSpA diagnosis was based on different types of criteria (NY, Amor, ESSG). Amor and ESSG criteria based diagnoses may include non-axial forms, which are known to be less associated with HLA-B27. The third heterogeneity is related to the control groups: absent in two-thirds of the studies, general rheumatology patients, blood donors. And finally, heterogeneity may be related to the method of HLA-B27 testing, which could not be assessed because it was often not described. Older testing methods may have been less sensitive.

## Conclusion

Since HLA-B27 is now a key feature in the diagnosis of SpA, the strength of its association with SpA in Middle-Eastern and Arab countries should be addressed in high quality studies: sufficient sample size, adequate diagnosis of SpA according to the latest criteria, adequate control group and sensitive HLA-B27 testing methods. It would be interesting to evaluate the performance of the ASAS criteria in our specific population. Furthermore, identification of new diagnostic markers in these specific populations is warranted.

## Abbreviations

AS: Ankylosing Spondylitis
AxSpA: Axial Spondyloarthritis
CI: Confidence Interval
ESSG: European Spondyloarthropathy Study Group
NY: New York
OR: Odds Ratio
SpA: Spondyloarthritis
